# Lipid Composition Affects the Efficiency in the Functional Reconstitution of the Cytochrome *c* Oxidase

**DOI:** 10.3390/ijms21196981

**Published:** 2020-09-23

**Authors:** Katharina Gloria Hugentobler, Dorothea Heinrich, Johan Berg, Joachim Heberle, Peter Brzezinski, Ramona Schlesinger, Stephan Block

**Affiliations:** 1 Institute of Chemistry and Biochemistry, Emmy-Noether Group “Bionanointerfaces”, Freie Universität Berlin, Arnimallee 22, 14195 Berlin, Germany; hugentobler@zedat.fu-berlin.de; 2 Department of Physics, Genetic Biophysics, Freie Universität Berlin, Arnimallee 22, 14195 Berlin, Germany; dorothea.heinrich@fu-berlin.de (D.H.); r.schlesinger@fu-berlin.de (R.S.); 3 Department of Biochemistry and Biophysics, The Arrhenius Laboratories for Natural Sciences, Stockholm University, SE-106 91 Stockholm, Sweden; johan.berg@dbb.su.se (J.B.); peterb@dbb.su.se (P.B.); 4 Department of Physics, Experimental Molecular Biophysics, Freie Universität Berlin, Arnimallee 22, 14195 Berlin, Germany; jheberle@zedat.fu-berlin.de

**Keywords:** proton translocation, single molecule, proton pump, electron transfer, membrane protein, single enzyme fluorescence microscopy

## Abstract

The transmembrane protein cytochrome *c* oxidase (C*c*O) is the terminal oxidase in the respiratory chain of many aerobic organisms and catalyzes the reduction of dioxygen to water. This process maintains an electrochemical proton gradient across the membrane hosting the oxidase. C*c*O is a well-established model enzyme in bioenergetics to study the proton-coupled electron transfer reactions and protonation dynamics involved in these processes. Its catalytic mechanism is subject to ongoing intense research. Previous research, however, was mainly focused on the turnover of oxygen and electrons in C*c*O, while studies reporting proton turnover rates of C*c*O, that is the rate of proton uptake by the enzyme, are scarce. Here, we reconstitute C*c*O from *R. sphaeroides* into liposomes containing a pH sensitive dye and probe changes of the pH value inside single proteoliposomes using fluorescence microscopy. C*c*O proton turnover rates are quantified at the single-enzyme level. In addition, we recorded the distribution of the number of functionally reconstituted C*c*Os across the proteoliposome population. Studies are performed using proteoliposomes made of native lipid sources, such as a crude extract of soybean lipids and the polar lipid extract of *E. coli*, as well as purified lipid fractions, such as phosphatidylcholine extracted from soybean lipids. It is shown that these lipid compositions have only minor effects on the C*c*O proton turnover rate, but can have a strong impact on the reconstitution efficiency of functionally active C*c*Os. In particular, our experiments indicate that efficient functional reconstitution of C*c*O is strongly promoted by the addition of anionic lipids like phosphatidylglycerol and cardiolipin.

## 1. Introduction

Living is hard work. The process of aerobic respiration is employed by all aerobic organisms to extract energy from environmental compounds in order to drive a wide range of processes such as motion, growth or signaling. In the last steps of this process, membrane-bound enzymes couple electron-transfer reactions to translocation of protons in order to generate and maintain an electrochemical gradient across membranes [1,2,3]. These gradients are then used by further enzymes to catalyze the synthesis of adenosine triphosphate, serving as a chemical energy source for subsequent biological processes [4].

Although the molecular components and architecture of cells employing aerobic respiration can vary considerably, the basic concept of this process is implemented in a highly conserved fashion [5]. For example, the enzyme cytochrome *c* oxidase (C*c*O) acts as terminal electron acceptor in the respiratory chain of many organisms employing aerobic respiration, including prokaryotic cells like bacteria and also mitochondria, which are the “power plants” of eukaryotic cells. Cytochrome *c* oxidase is a transmembrane protein that catalyzes the reduction of dioxygen to water and uses the energy obtained by this exogenic reaction to pump protons across the membrane [3,6]. The catalytic process of C*c*Os involves uptake and turnover of oxygen, electrons, and protons and can therefore be assessed in terms of the rates of electron transfer reactions, the rate of substrate oxidation, or proton uptake [3,7]. The catalytic center of the C*c*O is structurally highly conserved in different species [8], making C*c*O an established model enzyme to study the electron-mediated generation of a proton gradient across a lipid bilayer [3,9]. In contrast to the catalytic center, the lipid composition of the C*c*O containing membranes shows considerable variations among different species: Bacteria are often rich in phosphatidylethanolamine (PE) [10,11], while the lipid composition of mitochondrial membranes is typically dominated by phosphatidylcholine (PC) alone or a mixture of PC and PE [12]. Furthermore, a notable variation can also be observed for less abundant lipid species like phosphatidylglycerol (PG) and cardiolipin (CA), both of which have been shown to play crucial roles in stabilizing the energy converting machinery of bacterial cells and mitochondria [10,13,14,15]. For example, CA is known to interact with high affinity with a large number of proteins present in bacterial or mitochondrial membranes, including cytochrome *c* and C*c*O [16,17]. These interactions, typically mediated by hydrogen bonds to the phosphate groups of CA and van der Waals interactions to the acyl chains of CA [16,17], make CA an important structural constituent of the respiratory chain [18].

Since the activity of many enzymes is strongly dependent on the enzyme’s immediate environment [19,20,21], this raises the question to what extent the catalytic activity of C*c*O is affected by the lipid composition of the hosting membrane [22], in particular, as the known lipid binding sites of C*c*O also show high conservation across species [23]. Moreover, previous studies using a similar enzyme show an influence of membrane composition on enzymatic activity and proton translocation along the membrane [24,25]. Motivated by these considerations, we set out to investigate the influence of the lipid environment on the proton turnover rate of *R. sphaeroides* C*c*O. Inspired by recent studies on a related enzyme [26,27,28], this is achieved by reconstituting C*c*O into liposomes containing a pH sensitive dye and by probing changes of the pH value inside the proteoliposomes (caused by proton uptake by C*c*O). Analysing pH changes on the level of single proteoliposomes using fluorescence microscopy allows for the determination of single-enzyme kinetics and thus yields information on the proton turnover rate of single C*c*O in dependence of its lipid environment. The results are cross-validated using a cuvette-based spectrofluorometric assay [29], which provides an ensemble-averaged recording of luminal pH values. In comparison to the microscopy-based assay, this approach offers a higher signal-to-noise ratio in the recording process but does not permit a direct extraction of the rate of proton turnover.

## 2. Results and Discussion

**Figure 1:**
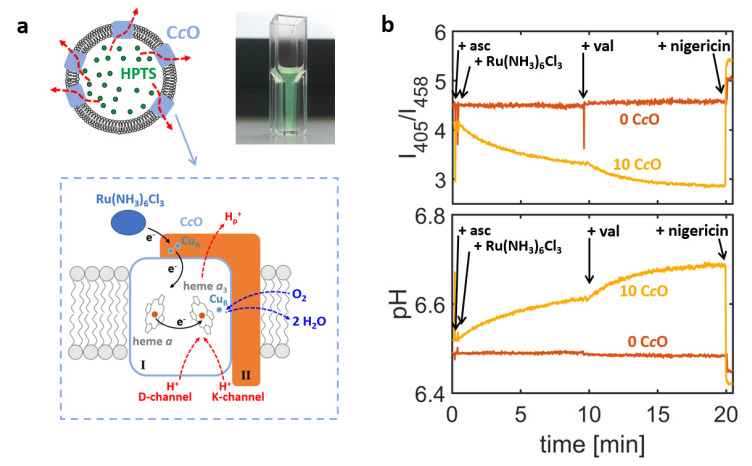
(**a**) Proton turnover of cytochrome *c* oxidase (C*c*O) is evaluated by reconstituting the enzyme into liposomes filled with the pH-sensitive dye 8-hydroxypyrene-1,3,6-trisulfonic acid (HPTS). The pH value inside liposomes is determined by recording the HTPS fluorescence emission at 520 nm for excitation at 405 nm or 458 nm, yielding the emission intensities *I*_405_ or *I*_458_, respectively, and converting the ratio *I*_405_/*I*_458_ (after calibration) into a pH value. Using a cuvette-based spectrofluorometer for readout yields emission intensities *I*_405_ and *I*_458_ averaged over many C*c*O-containing proteoliposomes. The resulting ensemble-averaged luminal pH value is obtained with high signal-to-noise ratio. A representative example is given in (**b**), showing changes of the luminal pH value of liposomes, either reconstituted with nominal 10 C*c*Os from *R. sphaeroides* per liposome (orange trace) or left empty (brown trace). Proton translocation is initiated after addition of ascorbate (asc) and substrate hexaammineruthenium chloride (Ru(NH_3_)_6_Cl_3_), while addition of the ionophores valinomycin (val) and nigericin (nig) quenches either the electric contribution (val) or the entire electrochemical gradient (val + nig). Note that ascorbate is not able to directly reduce C*c*O at the low concentrations used, but serves here as reducing agent for the ruthenium complex in order to keep the electron flow constant. Parameters of the experiment: C*c*O from *R. sphaeroides* reconstituted in liposomes generated using the crude extract of soybean lipids; Addition of 2 mM asc, 50 µM Ru(NH_3_)_6_Cl_3_, 2 nM val, 2 nM nig.

Similar to previous studies on the enzymatic activity of the *bo*_3_ quinol oxidase [28,29], a proteoliposome-based assay was used to quantify proton turnover of C*c*Os. To this end, C*c*Os were reconstituted into the membrane of large unilamellar vesicles (LUVs) and the lumen of formed proteoliposomes was filled with the pH sensitive dye 8-hydroxypyrene-1,3,6-trisulfonic acid (HPTS; Figure 1a) [30,31]. HPTS is a ratiometric dye and monitors the luminal pH value by (*i*) recording the fluorescence emission at 520 nm for excitation wavelengths of 405 nm and 458 nm, respectively, and (*ii*) calculating the ratio of the so-obtained emission intensities, *I*_405_/*I*_458_. The ratio of the intensities can be converted into the luminal pH value based on calibration experiments, in which HPTS was exposed to solutions of known pH values [28].

Enzymatic activity of the C*c*O-containing proteoliposomes was first assessed using a cuvette-based bulk spectrofluorometer [29], in which the emission intensities *I*_405_ and *I*_458_ are averaged over all proteoliposomes present in the optical readout volume. This approach yields ensemble-averaged information on the luminal pH value and therefore cannot be used for exact quantification of the proton turnover rates. Owing to the averaging process, this approach offers high signal-to-noise ratios and even minute changes of the luminal pH values are registered. A representative example is given in Figure 1b (orange trace). Here, changes of the intensity ratio *I*_405_/*I*_458_ and the corresponding ensemble-averaged pH values are shown for proteoliposomes formed by reconstituting *R. sphaeroides* C*c*O into liposomes made of a crude extract of soybean lipids, which is a lipid composition commonly used in functional studies of respiratory enzymes (see Table 1 for the lipid compositions used in this study) [32].

For these experiments, a concentration ratio of 10 C*c*Os per liposome was chosen during the reconstitution process, which means that the reconstitution mixture contained a molar concentration of C*c*O that was 10 times larger than the one of liposomes (calculated from the lipid content and using a mean liposome diameter of 150 nm, see Materials and Methods section).

Proton turnover by C*c*Os was initiated by addition of the electron-donor hexaammineruthenium chloride (Ru(NH_3_)_6_Cl_3_), which led to uptake of protons by the enzyme from the interior of the liposome and thus to an increase in luminal pH value. Although the enzyme can, in principle, be reconstituted in both orientations into the liposome’s membrane, only those enzymes with the electron-loading-site facing out of the liposome can be reduced by the ruthenium complex and are therefore active, while oppositely oriented enzymes cannot be reduced and remain inactive. Hence, in this assay the active enzyme’s N- and P-side are always oriented towards the liposome’s interior and exterior, respectively, and the catalytic activity of the C*c*O always causes the luminal pH value to increase.

This increase in pH value is typically accompanied by the generation of an electric potential across the liposome’s membrane [1], creating an electrochemical gradient, against which protons are taken up by C*c*O during the course of its catalytic cycle. This causes the luminal pH value to saturate after a certain time. Saturation is characterised by an equilibrium between proton uptake by the enzyme and proton permeation through the membrane (driven by the gradient in proton concentration). Addition of the potassium ionophore valinomycin selectively removes the electric contribution to the electrochemical gradient, which results in an increase of the C*c*O proton turnover rate and thus increases the pH value at saturation. As an internal control, the ionophore nigericin was added at the end of the experiment. The combined effect of nigericin and valinomycin renders the membrane permeable to protons, so that the luminal pH value is clamped to the pH value of the bulk solution. Therefore, a return to the initial pH value indicates that the action of the C*c*O indeed increases luminal pH value against the pH gradient generated. Thus, it is possible to distinguish between active proton translocation (against a pH gradient) and passive proton permeation (along a pH gradient) using this setup. As an additional control, the performance of C*c*O-containing proteoliposomes (Figure 1b, orange trace) was always compared to liposomes also filled with HPTS but without addition of enzyme in the reconstitution step (“empty liposomes”; Figure 1b, brown trace) and therefore showed no change in luminal pH value upon addition of reducing agents.

**Figure 2:**
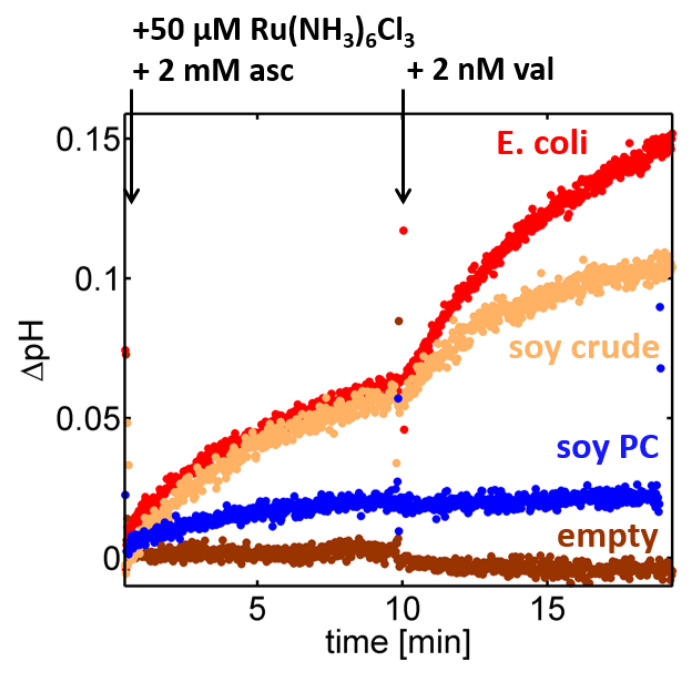
Proton translocation by *R. sphaeroides* C*c*Os reconstituted into liposomes composed of either the polar extract of *E. coli* (red), a crude lipid fraction of soybean-derived lipid (orange), or the PC fraction of soybean-derived lipids (blue). In all cases, C*c*O was reconstituted at a nominal concentration ratio of 10 enzymes per liposome and the change in the luminal pH value was determined using the cuvette-based spectrofluorometer setup (Figure 1). Data from liposomes lacking C*c*O (“empty”) were added for comparison (brown; crude lipid fraction of soybean).

In order to probe the impact of the lipid composition, related experiments were conducted using proteoliposomes formed through reconstitution of the enzyme into either the polar extract of *E. coli*, a lipid composition often used in the reconstitution of heme-copper oxidases [26,28], or into soybean PC, which is the major constituent of the soybean crude extract. For C*c*O reconstituted in a crude lipid fraction of soybean lipids, the proton turnover saturated at a pH gradient of ~0.07 pH units prior to valinomycin addition and at ~0.1 pH units afterwards (cf. Figure 2, orange trace). Surprisingly, the saturation values dropped considerably when a lipid composition consisting of 95% soybean phosphatidylcholine (PC) was used instead (Figure 2, blue trace), while a similar performance was observed using the polar extract of *E. coli* (Figure 2, red trace). Although in all cases the nominal concentration ratio of enzymes per liposome was fixed at 10:1 in the reconstitution step, changes in the lipid composition generated different pH traces in the spectrofluorometric setup, which is indicative of an impact of the lipid composition on either the reconstitution efficiency of C*c*Os (number of active enzymes per liposome), the proton turnover rate of single C*c*O, changes in the proton permeability of the liposomes, or combinations of these effects.

**Figure 3:**
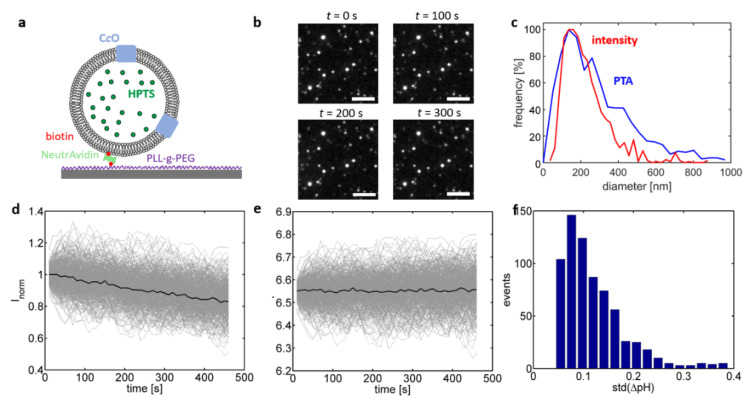
(**a**) C*c*O-containing liposomes (nominal 3 C*c*O per liposome; crude fraction of soybean lipids) were immobilised to a glass surface by cross-linking biotinylated lipids with biotinylated PLL-g-PEG copolymers using NeutrAvidin. (**b**) Image of immobilised C*c*O-containing liposomes (excitation at 458 nm, emission recorded at 520 nm = *I*_458_) at different time points as indicated (scale bar = 6.5 µm). *I*_458_ shows a large variation among the proteoliposomes, which is caused by the broad size distribution of the proteoliposomes. (**c**) Size distributions of the C*c*O-proteoliposomes determined using particle tracking analysis (PTA; blue) of bulk-dissolved proteoliposomes and by taking the third root of the initial *I*_458_ value (red). (**d**) Time-dependent *I*_458_ traces after normalisation by their starting value (grey lines) and its ensemble-averaged value (black line). Without substrate addition the normalised *I*_458_ intensity follows an exponential decay caused by bleaching of HPTS. (**e**) Bleaching can be accounted for by determining the bleaching rate of the ensemble average and correcting the *I*_458_ intensity traces accordingly. After conversion into pH values, the corresponding pH traces fluctuate, in absence of proton translocation, around their initial value. (**f**) Distribution of the standard deviation of the pH traces indicates that the accuracy in the determination of luminal pH values is better than 0.1 pH units.

Although the cuvette-based assay supports rapid determination of such differences between different lipid compositions, it cannot be used on its own to determine the C*c*O proton turnover rate. As the proteoliposomes show a notable variation in size and number of (actively) reconstituted enzymes, the ensemble-averaged pH trace is a superposition of several proteoliposome populations and thus depends in a complex way on various parameters. In light of this finding, probing luminal pH values on the level of single liposomes using fluorescence microscopy proved to be a potent approach to analyze the activity of single proton pumps [26,27,28]. In this approach, the proteoliposomes are linked to a glass interface and fluorescence microscopy is used to quantify the individual change in fluorescence emission of single, interface-bound proteoliposomes. This concept was realised in the present study by complementing the lipid composition of the liposomes with DPSE-PEG-biotin lipids, which allowed for linking the biotinylated proteoliposomes to a biotinylated glass interface by means of the highly specific biotin-(Neutr)Avidin interaction (Figure 3a) [28]. The luminal pH value of single, isolated liposomes was determined through excitation of HPTS present inside the liposomes at 458 nm and imaging the fluorescence emission of the interface-bound liposomes at 520 nm.

In the first set of experiments (Figure 3; C*c*O reconstituted into a crude extract of soybean lipids), changes in the emission intensity *I*_458_ over time were determined in the absence of reducing agents, i.e., without activation of the enzyme. Isolated proteoliposomes were adequately resolvable by fluorescence microscopy. Variance of the emission intensities between proteoliposomes is attributed to notable variations in the proteoliposome size within the ensemble. This hypothesis was confirmed by performing particle tracking analysis (PTA) of bulk-dissolved proteoliposomes, which yielded a broad distribution of the proteoliposome’s hydrodynamic diameter (Figure 3c, blue trace; mode at approximately 150 nm). As an increase in the proteoliposome size also increases the proteoliposome volume and thus the number of encapsulated HPTS molecules, the emission intensity scales to the third power with the proteoliposome size [33], which explains the observed variation of the proteoliposome intensity in Figure 3b. This relationship also provides a means to determine in situ the size of an imaged proteoliposome by taking the third root of its initial emission intensity. The unknown prefactor used in the conversion of intensity to size is obtained matching the modes of intensity- to PTA-derived size distribution (Figure 3c) [33]. Beyond obtaining the size distribution of the sample, this approach also offers the possibility to selectively include only proteoliposomes with a defined size range, which are therefore equivalent with respect to size-dependent properties such as the buffering capacity. This is an advantage in comparison to spectrofluorometric recordings that average over all proteoliposomes present in the optical readout volume. In the following discussion the analysis of single-proteoliposome measurements was restricted to proteoliposome sizes within 75–125% of the mode value, which typically covered >50% of the entire ensemble.

Extracting *I*_458_ traces recorded in the absence of reducing agents yielded exponentially decaying curves, which were attributed to bleaching of HPTS molecules during imaging (Figure 3d). In order to quantify bleaching, the ensemble average was calculated after normalizing all *I*_458_ by their initial intensity (black line in Figure 3d), followed by dividing this ensemble average from each *I*_458_ trace. Converting the bleaching-corrected *I*_458_ traces into pH values yielded pH traces that randomly fluctuated around their initial value (Figure 3e), which is expected as no substrate was added. These fluctuations are indicative of the accuracy, with which changes in the luminal pH value can be determined on the level of single proteoliposomes. Calculating the standard deviation for each pH trace showed that this random fluctuation was below 0.1 pH units for most pH traces.

**Figure 4:**
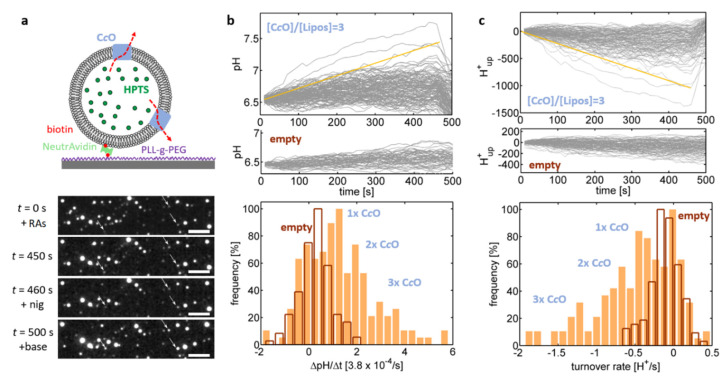
(**a**) C*c*O-containing liposomes (nominal 3 C*c*O per liposome; crude fraction of soybean lipids) were immobilised and recorded as described in Figure 3. Proton translocation was initiated at *t* = 0 s by addition of the reducing agents (RAs) ascorbate and Ru(NH_3_)_6_Cl_3_, causing the luminal pH value to increase (see white arrows for representative examples). As internal control, nigericin was added at *t* = 460 s to equilibrate the generated pH gradient. Furthermore, at *t* = 500 s the buffer outside the liposomes was exchanged from pH = 6.5 to 7.15, allowing for probing the pH readout of the proteoliposomes. (**b**, **top**) pH traces of liposomes containing and devoid of C*c*O, respectively. (**b**, **bottom**) The distribution of the slope of the pH traces ΔpH/Δ*t* (yellow line) fluctuates around 0 for empty liposomes but exhibits distinct peaks for C*c*O-containing proteoliposomes, which are attributed to be populations containing no, one, two, and three pumping-competent C*c*O per liposomes (as indicated). (**c**) The pH traces allow for extracting the number of protons, H^+^_up_, that are taken up by C*c*O to yield the observed pH traces. The slope of these traces corresponds to the total proton turnover rate of the proteoliposomes, the distribution of which also shows distinct peaks.

In the next set of experiments, the same proteoliposomes were used to probe proton uptake by C*c*Os (Figure 4). The enzyme was activated by addition hexaammineruthenium chloride and ascorbate and the resulting increase of the luminal pH value was again quantified by the increase in the *I*_458_ emission intensity (Figure 4b). Some of the pH traces fluctuated, similar to Figure 3e, around their initial value, which is indicative of lack of proton uptake by the enzyme, while other traces showed a notable increase in luminal pH values (Figure 4b, top plot). This population was absent in liposomes where no enzyme was added in the reconstitution step of the preparation (“empty liposomes”; Figure 4b, middle plot) and is therefore indicative of the presence of functionally reconstituted enzymes in this population. This interpretation was further supported by addition of nigericin at *t* = 460 s, which recovered the luminal pH to its initial value (= the external pH value), thereby proving proton uptake by the C*c*O against the action of the established pH gradient. As the pH traces increased linearly over time, the corresponding slope, ΔpH/Δ*t* was determined based on linear fits applied to the pH traces. The distribution of these slopes (Figure 4b, bottom plot) exhibited 4 peaks, which were attributed the presence of four populations corresponding to empty liposomes and proteoliposomes being equipped with one, two and three pumping-competent C*c*Os, respectively.

In order to determine C*c*O proton turnover rates for the obtained pH traces, we modelled the proteoliposomes as spheres with known diameter (determined by PTA measurements). Proton efflux out of the sphere (caused by proton uptake of C*c*O), results in a decrease in the proton concentration within the proteoliposome, which is partially compensated by deprotonation of the buffer molecules according to the Henderson–Hasselbalch-equation [34]. As the p*K*-values of MOPS and HPTS are almost identical (7.2 vs. 7.3), we did not discriminate between these two buffer populations. Furthermore, the rate of proton dissociation from buffer molecules is expected to be much faster than the rate of proton efflux, which allows to decouple the equations describing proton efflux and proton dissociation.

The proteoliposome buffer capacity was determined by passive permeation measurements as described in the Materials and Methods section, while their size was determined by matching intensity- and PTA-derived size distributions (Figure 3c). Using this information allowed to numerically calculate the relationship between total proton concentration (free + buffer bound) inside the proteoliposomes and the exhibited luminal pH. Thereby we determined, how many protons were taken up by CcO in order to explain the observed changes of the luminal pH values (Figure 4c, top plot). Mirroring the pH traces, these traces showed a linear time dependence and their slope therefore corresponds to the rate of proton efflux from the proteoliposome (i.e., the rate of proton uptake by the enzyme), which can be extracted by application of linear fits. The distribution of these rates exhibited several equidistant peaks occurring at multiples of ~0.4 H^+^/s (Figure 4c, bottom plot; see Figure S2, for more examples), which were again attributed the presence of four populations corresponding to empty liposomes and proteoliposomes being equipped with one, two and three C*c*O(s), respectively. Under the conditions employed in this assay, a single C*c*O reproducibly takes up ~0.4 H^+^/s, which is several orders of magnitude below the maximum proton turnover rate typically reported for C*c*O [35]. This observation indicates that the enzymes are not operating at saturation of the proton turnover rate, i.e., the proton uptake rate is limited by the delivery of electrons by the substrate (hexaammineruthenium chloride). This reasoning is in agreement with the observations that increasing the substrate concentration also increases the proton uptake rate by the proteoliposomes. Furthermore, measurements on the oxygen consumption of the C*c*Os in excess of the native substrate, cytochrome *c*, yielded an electron turnover rate of 910 ± 50 electrons/s (*n* = 4) at pH = 6.5, which is close to typically reported values (~1000 electrons/s) [36], while almost no electron turnover was detected using hexaammineruthenium chloride as substrate. These findings indicate that hexaammineruthenium chloride is limiting the enzymatic turnover while the enzyme activity itself is not disturbed *per se*.

**Figure 5:**
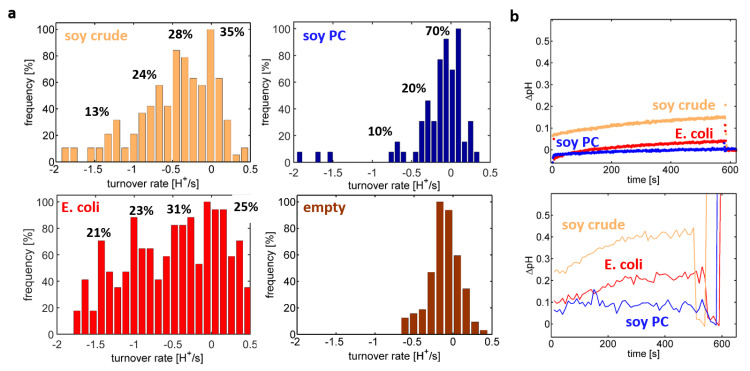
(**a**) Distributions of the total proton turnover rate indicate that the lipid composition strongly affects the efficiency of reconstitution of C*c*O but has only a minor effect on the proton turnover rate of single C*c*Os. Data from liposomes lacking C*c*O were added for comparison (brown; crude lipid fraction of soybean). (**b**) Comparison of ensemble-averaged pH traces obtained using a cuvette-based spectofluorometer (top) or by averaging the pH-traces obtained from the single-liposome assay (bottom).

In order to understand the reason for the differences observed in the cuvette-based assay, the single-liposome assay was applied to proteoliposomes differing in their lipid composition (Figure 5a). Comparable proton turnover rate distributions were extracted for a crude extract of soybean lipids and the polar extract of *E. coli*, both exhibiting four peaks at multiples of ~0.4 H^+^/s, which were attributed to populations containing between zero and three functionally active C*c*Os per liposome. Although the proteoliposomes were formed using a mixture containing a concentration ratio of three enzymes per liposome, on average only approximately one C*c*O was functionally reconstituted per liposome for both lipid compositions. Thus, we can infer that the liposomes had on average only one pumping-competent C*c*O reconstituted in the membrane on average, although three C*c*O per liposome were present in the reconstitution mixture. In contrast, proton turnover rate distributions of soybean PC-based proteoliposomes showed (in addition to the inactive population at 0 H^+^/s) only a single clearly resolvable peak at ~0.4 H^+^/s and indications of a second peak at ~0.8 H^+^/s. Here, an even lower ensemble average value of ~0.3 functionally reconstituted C*c*Os per liposome is obtained.

In all of these cases, the average number of functionally reconstituted C*c*Os was smaller than expectation based on the concentration ratio of C*c*O versus proteoliposomes in the reconstitution step. These findings imply that only a fraction of available enzymes is reconstituted into the liposomes and/or that only a fraction of the reconstituted C*c*Os is correctly oriented, so that the electron loading site is accessible to the substrate. The proton turnover rate distributions of Figure 5a showed only non-significant variations in the position of the peak values but differed mainly in the number distribution of functionally reconstituted CcOs. This implies that the differences observed in the spectrofluorometric assay (Figure 2) are mainly caused by an impact of the lipid composition on the efficiency in functional reconstitution of C*c*O.

In order to test, whether the single-liposome data is representative of the ensemble probed by the spectrofluorometric assay, the ensemble-averaged pH trace was determined by averaging all single-liposome pH traces included in the analysis (Figure 5b). Indeed, the lipid composition-dependent behaviour of proteoliposome preparations in the microscope assay (Figure 5b, bottom) resembled the behaviour observed in the cuvette-based spectrofluorometer assay (Figure 5b, top). Both approaches yielded qualitatively similar results but scaled by factor of ~2. This is attributed to the fact that the single-liposome assay was restricted to proteoliposome sizes being close to the mode of the entire distribution, so that the average buffer capacity in the single-liposome assay was smaller than the one of the spectrofluorometric assay. Calculating the ensemble average without using a constraint on the proteoliposome size yielded pH traces approaching the ones of the spectrofluorometer assay (data not shown).

The data shown in Figure 5 demonstrate that PC alone does not allow for efficient reconstitution of C*c*O. This is indicative of a promoting effect of one -or more- lipids present in the native compositions tested so far. The lipid composition of *E. coli* polar extract is well described and consists of 66.7 wt% PE, 23.1 wt% PG and 9.8 wt% CA (cf. Table 1, row 3), which compares reasonably well with the lipid composition of *R. sphaeroides* (e.g., strain 2.4.1: 40 wt% PE, 11 wt% PG, 12 wt% CA, 57 wt% other lipids) [13]. It was therefore used as a reference composition to study the influence of its lipids on C*c*O reconstitution efficiency. In order to probe the influence of each lipid, a synthetic version of *E. coli* polar extract was sequentially approached replacing PC with the respective amounts of PE, PE + PG, and PE + PG + CA (for compositions of complex synthetic lipid compositions cf. Table 1 rows 4–6).

**Figure 6:**
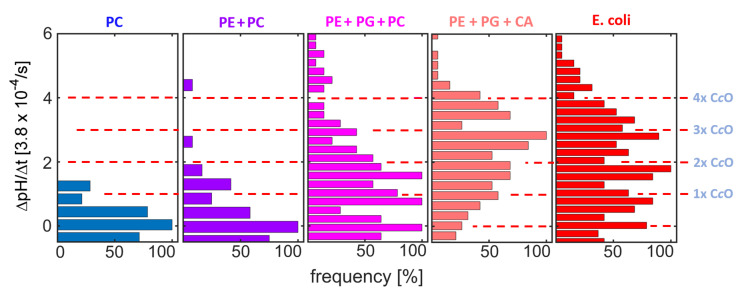
ΔpH/Δ*t* distributions of C*c*O-containing liposomes using the lipid compositions indicated (see Table 1 for exact numbers of the supplemented lipid species). Although the concentration ratio of enzyme to liposome was fixed at 3:1 in all cases (i.e., nominal 3 C*c*O per liposome were present during the reconstitution process), the amount of functionally reconstituted enzyme differs strongly among the different lipid compositions tested here.

Pumping was observed for all measurements using the synthetic lipid compositions. A linear increase in pH vs. time, similar to the one reported in Figure 4, allowed for determining the rate of pH change (ΔpH/Δt). The ΔpH/Δt distributions obtained here (Figure 6) show equidistant peaks for most lipid compositions. The positions of the peaks reproduce well among compositions (red dashed lines in Figure 6). As in the experiments employing native compositions reported above (Figure 4), these peaks are attributed to proteoliposome subpopulations with different numbers of functionally reconstituted C*c*Os (as indicated at the red dashed lines in Figure 6). Comparing the protein distributions between the synthetic compositions, a clear impact of PG and CA on the efficiency of functional reconstitution is observed, while addition of PE caused only minor changes to the distribution.

Considering that PC and PE are neutral, while PG and CA are negatively charged at physiological pH, this finding suggests electrostatic interactions to be important for cholate-based reconstitution of C*c*O. Such interactions were already shown to be very important for the high affinity interactions formed between CA and respiratory chain proteins like C*c*O [16,17], and are thus likely to promote the incorporation of the enzyme into CA-containing membranes. Furthermore, recent investigations have shown that depletion of CA in *R. sphaeroides* induces a remodelling of the bacterial membrane’s lipid composition, in which CA gets substituted by anionic lipids like PG [10,13]. As a consequence of this remodelling, CA depletion does neither affect C*c*O structure nor function, further indicating that negatively charged lipid species can substitute at least part of the function of CA in *R. sphaeroides*.

## 3. Materials and Methods

All reagents unless otherwise indicated were bought from Sigma Aldrich (Steinheim, Germany) in the highest purity grade available. The following lipids were used for formation of liposomes: a crude extract of soybean (P5638, Sigma-Aldrich), the PC fraction of soybean lipids (95% purity; order number 441601P, Avanti Polar Lipids, Alabaster, AL, USA), the PE fraction of soybean lipids (>99% purity; order number 840024P, Avanti Polar Lipids), the PG fraction of soybean lipids (>99% purity; order number 841148P, Avanti Polar Lipids), the polar extract of *E.coli* (order number 541601P, Avanti Polar Lipids) and *E. coli* cardiolipin (order number 841199P, Avanti Polar Lipids).

### 3.1. Buffers

*Assay buffer*: MOPS (10 mM), K_2_SO_4_ (50 mM), at either pH 6.5, pH 7.15 or 7.9. *Assay master mix*: Assay buffer pH 6.5 plus valinomycin (2 nM), ascorbate (2 mM) and hexaammineruthenium(III) cloride (50 μM). *Detergent buffer*: Sodium phosphate buffer (50 mM), n-dodecyl-β-D-maltoside (2%), PMSF (1 mM), pH 8. *Reconstitution buffer*: MOPS (2 mM), K_2_SO_4_ (50 mM), pH 6.5. *Resuspension buffer*: Sodium phosphate buffer (50 mM), MgCl_2_ (5 mM), pH 8. *Wash buffer A*: Sodium phosphate buffer (50 mM), imidazole (10 mM), DDM (0.1%), KCl (200 mM), pH 8. *Wash buffer B*: Sodium phosphate buffer (250 mM), imidazole (15 mM), DDM (0.1%), KCl (200 mM), pH 8. *Wash buffer C*: Sodium phosphate buffer (250 mM), imidazole (25 mM), DDM (0.1%), KCl (200 mM), pH 8. *Elution buffer*: Sodium phosphate buffer (50 mM), imidazole (100 mM), DDM (0.1%), KCl (200 mM), pH 8.

### 3.2. Stock Solutions

(+)-Sodium L-ascorbate was dissolved in deionised water to yield a 600 mM solution, distributed in 50 μL aliquots and stored at −20 °C. For measurement, 24.6 μL assay buffer was added to one aliquot to yield a 402 mM stock solution. Sodium cholate hydrate was dissolved in deionised water to yield a 580.7 mM solution and distributed in 15–20 μL aliquots. The aliquots were stored at −20 °C and individually thawed for each reconstitution. Hexaammineruthenium (III) chloride was dissolved in deionised water to yield a 306 mM solution, distributed in 3.3 μL aliquots and stored at −20 °C. For measurement 96.7 μL assay buffer was added to one aliquot to yield a 10.1 mM stock solution. HPTS was dissolved in deionised water to yield a 100 mM solution, distributed in 250 μL aliquots and stored in the dark at −20 °C. Nigericin was dissolved in ethanol to yield a 1μM solution and stored at −20 °C. Valinomycin was dissolved in ethanol to yield a 1μM solution and stored at −20 °C.

### 3.3. Media

Culture medium: Sistrom’s succinate-based minimal medium was supplemented with streptomycin (68 μM), spectinomycin (100 μM), and tetracycline (2.3 μM) [37].

### 3.4. Homologous Expression and Purification of CcO from Rhodobacter Sphaeroides

The *R. sphaeroides* strain JS100 containing the genetic information of subunits I-IV of the *aa*_3_-type C*c*O (WT) (with an additional C-terminal 6xHis tag on subunit I) were cultivated under aerobic conditions. The main culture was grown on Sistrom’s succinate-based minimal medium at 30 °C for 36 h and aeration through shaking at 120 rpm. To harvest, the culture was centrifuged at 7450× *g* for 20 min and the pellet resuspended in resuspension buffer to yield a concentration of 0.5 g cells/mL. To purify the protein, DNase and 1 mM PMSF were added to the buffer prior to cell disruption by 2–4 passages through a Cell Disrupter TS 1.1 (Constant Systems Ltd., Low March, Daventry Northants, UK) at 1.7 kbar pressure. 

The membranes with C*c*O were harvested at 244,000× *g* for 2 h and resuspended in detergent buffer (1 g membrane pellet/5 mL buffer). To solubilize C*c*O, the solution was stirred overnight at 4 °C. The extract was clarified by centrifugation at 244,000× *g* for 2 h to remove any remaining insoluble material. The pure protein was isolated through affinity chromatography via its 6xHis-tag on a Ni-NTA Agarose affinity column (Protino Ni-NTA column, Macherey&Nagel, Germany). The enzyme was loaded onto a column filled with Ni^2+^ NTA resin. Subsequently the bound enzyme was washed with 15 column volumes wash buffer A, 10 column volumes wash buffer B, 7.5 column volumes wash buffer C, and a final 50 column volumes wash buffer A. The enzyme was eluted with 5 × 1 column volumes of elution buffer. Fractions containing the enzyme (fractions showing a dark green colour) were pooled and concentrated, and the buffer was exchanged to sodium phosphate buffer (50 mM) substituted with 0.1% DDM. 

Comparison of reduced and oxidised C*c*O absorption spectra following the protocol of van Gelder et al. [38] yielded a protein concentration of 90 μM.

### 3.5. Cytochrome c Oxidase Activity 

The C*c*O activity was measured using a Clark-type oxygen electrode (Hansatech, Norfolk, UK) operating at 25 °C. The reaction cell contained 50 mM KH_2_PO_4_ pH 6.5, 0.05% DDM, 1 mg/mL asolectin, 2.8 mM ascorbate, 0.55 mM TMPD, 30 μM of equine heart cytochrome *c* and 5 nM of the purified C*c*O. The C*c*O activity was calculated as outlined in Thompson et al. [39].

### 3.6. Lipid Preparations

Lipids were dissolved in CHCl_3_. DSPE-PEG-biotin (as a 0.04% (*w/v*) CHCl_3_ solution) was added at a lipid ratio of 0.04% (*w/w*). The lipid mixture was extensively dried (nitrogen flow and subsequent removal of residual CHCl_3_ on a membrane pump for >1.5 h). The dried lipids were resuspended in assay buffer at a lipid concentration of 10 mg/mL and subjected to 4 cycles of vortexing, snap-freezing in liquid N_2_, and thawing in water at room temperature. The preparations were stored at −80 °C until further use.

### 3.7. Reconstitution of CcO in Liposomes

The lipid preparation (225 μL per (proteo)liposome batch) was thawed, loaded in a mini extruder (Avanti Polar Lipids, Alabaster, AL, USA), and extruded 31× (200 nm Whatman PC filter). The preparation was distributed in Eppendorf tubes (225 μL per tube) and 6.25 μL cholate stock solution, 0.67 µL C*c*O stock solution, and 18.08 µL reconstitution buffer were added to yield a protein-liposome mixture having a nominal concentration ratio of 3 proteins per liposome (calculated for liposome diameter of 150 nm; see Figure S1, while adding 2 µL C*c*O stock solution, and 16.75 µL reconstitution buffer instead yielded a protein-liposome mixture with a nominal concentration ratio of 10 C*c*Os per liposome. As a control, “empty” liposomes were generated by adding the corresponding amount of buffer instead of protein. The reconstitution mixture was stored on ice for 30 min and was gently shaken every 10 min. Afterwards, each reconstitution mixture was loaded on a separate PD-10 column (GE Healthcare) that was pre-equilibrated with 25 mL deionized water and 25 mL reconstitution buffer. Subsequently, reconstitution buffer was loaded dropwise (750 μL followed by 2 × 950 μL). The proteoliposomes were eluted with reconstitution buffer (2 × 675 μL). HPTS stock solution (62.5 μL) was added to each proteoliposome solution and each mixture was subjected to 3 cycles of vortexing, snap-freezing in liquid N_2_, and thawing in water at room temperature. 1000 μL of each proteoliposome solution was again loaded onto the center of a PD-10 column (GE Healthcare) that was pre-equilibrated with 25 mL deionized water and 25 mL reconstitution buffer. Reconstitution buffer (2 × 950 μL) was added dropwise and subsequently, the proteoliposomes were eluted with reconstitution buffer (2 × 600 μL). The proteoliposomes were kept on ice and used for measurement on the day of preparation.

### 3.8. Bulk Spectrofluorometer Assay

Freshly prepared proteoliposomes were diluted 50× (20 μL in 980 μL assay buffer) in a 1000 μL in cuvette. To start the reaction 5 μL ascorbate and 5 μL hexaammineruthenium (III) chloride stock solutions were added. The reaction was mixed thoroughly by pipetting. After 10 min, 2 μL valinomycin stock solution was added to equilibrate the electric potential across the membrane. After additional 10 min, 2 µL nigericin stock solution was added to equilibrate the electric potential and proton gradient across the membrane. Measurements were recorded over >20 min on a Cary Eclipse Fluorescence Spectrophotometer (Agilent Technologies Sales & Services GmbH & Co.KG, Waldbronn, Germany; Software: Cary Eclipse Kinetics Application; V1.2(146)) using excitations at 405 nm (5 nm slit) and 458 nm (5 nm slit), respectively, and recording the emission at 510 nm (5 nm slit, 600 V gain at the PMT).

### 3.9. Microscopy-Based Single-Liposome Assay

Unless otherwise indicated all microscopy measurements were performed on a Nikon Eclipse Ti-E microscope (Nikon, Düsseldorf, Germany) equipped with a 100× Plan-Apo oil immersion objective (NA 1.45), a Lumen 200 (Prior Scientific, Cambridge, UK) white light source and an Andor Zyla 4.2 sCMOS camera (Oxford Instruments, Oxford, UK). All measurements were done using a ND32 neutral density intensity filter, 2 × 2 binning of the sCMOS camera, and using a dichroic filter set consisting of a 460 nm excitation filter (14 nm bandwidth), 495 nm dichroic filter, and a 460 nm emission filter (5 nm bandwidth).

Size determination by particle tracking analysis-Particle tracking analysis (PTA) was performed to determine the size of the different liposome preparations. Liposomes were diluted by factor 100 in assay buffer pH 6.5 and injected in a home-made PDMS stamp providing 4 wells (5 μL volume each) mounted onto a coverslip (#1; Menzel-Gläser, Braunschweig, Germany). Each sample was measured over 200 frames at an acquisition rate of 16.6 frames per second and at least 5 independent movies were recorded. The suspended liposomes were tracked using single particle tracking as described in Müller et al. [40] and the hydrodynamic radii were determined from the measured bulk diffusion coefficient using the Stokes-Einstein relationship. 

Surface functionalization-Glass cover slips (#1; Menzel-Gläser, Braunschweig, Germany) were cleaned according to the RCA-1 protocol and thoroughly rinsed with deionised water [28,41]. A home-made stamp based on polydimethylsiloxane (PDMS) provided 6 wells (5 μL volume each) and was mounted onto the cleaned coverslip. The glass interfaces were functionalized by incubation of the wells with a mixture of PLL-g-PEG and PLL-g-PEG-biotin block-copolymers (ratio: 2000:1 in assay buffer; total polymer concentration: 10^−3^ g/L; SuSoS AG, Dübendorf, Switzerland) for 10 min, followed by thoroughly rinsing with assay buffer, incubation with NeutrAvidin (50 µg/mL in assay buffer; Thermo Fisher Scientific) for 5 min and again thoroughly washing with assay buffer. The liposome sample was diluted by a factor of 1:166 in the assay buffer (1:20 for PC-based liposomes) and incubated until the surface was sufficiently populated (typically after 3 min of incubation). The supernatant was removed, and the sample washed with assay buffer prior to measuring.

Determination of the bleaching rate-Liposomes were immobilised at a glass interface (for preparation see sample preparation above). Bleaching was recorded over a time course of 100 s (1 s exposure, 2 s interval, 50 frames) without addition of any substances.

Single CcO measurements-Liposomes were immobilised at a glass interface (for preparation see sample preparation above). The reaction was started through buffer exchange to assay master mix and the pumping recorded for 8.3 min (1 s exposure, 10 s interval, 50 frames). Afterwards, the buffer was exchanged to assay buffer containing 2 nM nigericin for 5 frames and finally to assay buffer at pH 7.9 (w/o nigericin). The resulting microscopy movies were corrected for drift and the intensity of the liposomes were extracted as described in Berg et al. [28].

Determination of the permeation rate-Liposomes were immobilised at a glass interface (for preparation see sample preparation above). The reaction was started by buffer exchange to assay buffer pH 7.9 (supplemented with 2 nM valinomycin), which causes, due to permeation of protons along the pH gradient, an increase in the luminal pH value of the liposomes over time. This permeation process was recorded for 8 min (1 s exposure, 10 s interval, 50 frames).

Data analysis-The resulting microscopy movies of surface-linked (proteo)liposomes were corrected for drift and the intensity of the liposomes were extracted as described in Berg et al. [28]. The size of the imaged liposomes was determined by (*i*) extracting the intensity distribution of the first 5 frames (i.e., without notable bleaching of the sample and prior to addition of any substance), (*ii*) by calculating the third-root of this distribution yielding a size distribution with a yet unknown scaling factor (translating size into intensity) and (*iii*) by determining this pre-factor by matching PTA-measured and intensity-extracted size distributions [33]. As the liposome size has a strong impact on its buffering capacity, further analyses were restricted to liposomes having a size close to the mode of the size distribution (i.e., having a size within 75% to 125% of the mode value). The extracted intensity traces were translated into time-dependent (luminal) pH value traces using the known relationship between pH value and *I*_458_ intensity of HPTS (determined by calibration measurements as described in Berg et al. [28]).

For all lipid compositions, the buffering capacity was determined by generating proteoliposomes having a luminal concentration of 2 mM MOPS, as well as batches having only 0.2 mM and 0.02 mM MOPS instead. For each of these 3 batches, permeation experiments were performed as described above, yielding information on the time-dependent increase in luminal pH that is caused by exposing the liposomes to a pH gradient of 1.4 pH units (initial luminal pH value = 6.5 vs. pH = 7.9 of the measurement buffer). The rate of pH change depends on the pH gradient (which can be measured from the HPTS fluorescence), the proton permeation rate through the lipid membrane, and the luminal buffering capacity of the (proteo)liposome [42]. We described luminal buffering effects using a Henderson-Hasselbalch equation employing a pK_a_-value of 7.2, motivated by the values of MOPS (7.2) and HPTS (~7.3) and numerically solved the transport equation describing the permeation of protons through the membrane. Under the reasonable assumptions that the proton permeation rate does not depend on the buffer concentration and that the change in buffering capacity between (proteo)liposomes of different batches is given by the change in the luminal MOPS concentration, the buffer capacity as well as the proton permeation rate can be extracted from such measurements. While for soybean crude extract and soybean PC (95%) the buffering capacity is as expected from the luminal concentrations of MOPS and HPTS, we noticed a slightly larger buffering capacity for the polar extract of *E. coli* being 0.45 mM larger than the sum of the MOPS and HPTS concentration. All extracted permeation rates were on the order of ~ 10^−5^ cm/s (soybean crude extract: (0.78 ± 0.38) 10^−5^ cm/s, soybean PC: (0.90 ± 0.56) 10^−5^ cm/s, E. coli polar extract: (0.75 ± 0.71) 10^−5^ cm/s) and thus comparable to previously reported values [42,43].

Determining the buffering capacities of the different lipid compositions allowed for extracting the number of protons taken up by C*c*O in the single-liposome pumping assay. The analysis of these measurements again started with the extraction of the liposome size distribution as described above and by restricting further analyses to liposomes having a size close to the mode of the size distribution (i.e., having a size within 75–125% of the mode value). For each of these liposomes, the luminal pH value was extracted from the change in HPTS fluorescence intensity, yielding a set of time-dependent pH value traces (one trace for each tracked liposome). For each of these pH value traces, the Henderson-Hasselbalch equation (employing the buffering capacity determined as described above) was solved numerically in order to determine the number of protons that had been taken up by the oxidase to yield the observed change in luminal pH value.

*Measurement statistics*–Each lipid composition was characterised by at least 3 independent bulk spectrofluorometer measurements and 3 independent runs of the single-liposome assay. These replications were distributed over independent measurement days and done using freshly prepared proteoliposomes.

## 4. Conclusions

In this study the enzyme cytochrome *c* oxidase (C*c*O) from *R. sphaeroides* was reconstituted into the membranes of large unilamellar vesicles. Uptake of protons by the enzymes, which is part of its catalytic activity in order to reduce dioxygen to water, caused a change of the pH value inside of the proteoliposomes. This allowed for assessing the dynamics of proton uptake, that is, the rate of proton turnover by the enzyme. Applying fluorescence microscopy, single-liposome resolution was achieved in these measurements, yielding information on the distribution of proton turnover rates across the proteoliposome ensemble. These distributions typically exhibited several distinct peaks, which were attributed to liposome populations differing in the number of functionally reconstituted C*c*Os. It is shown that changing the lipid composition of the proteoliposomes has a strong impact on these distributions: While the investigated lipid compositions did not significantly affect the proton turnover rate of the single C*c*O, a strong effect on the distribution of functionally reconstituted C*c*O was observed. Most C*c*O was functionally reconstituted in lipid compositions from native sources, such as a crude extract of soybean lipids and the polar extract of *E. coli* were employed. For the latter composition, it is shown that the presence of the (at physiologically pH values) negatively charged lipids PG and CA are more important for a functional reconstitution of C*c*O than the neutral lipids PC and PE. Whether these differences are caused by an overall reduction in reconstitution efficiency of the enzyme or, for example, by a change in the partition between the two possible C*c*O orientations, requires further experiments. Nevertheless, a microscopy-based assessment of the enzymatic activity provides a means to assess the efficiency by which enzymes are functionally reconstituted into particular lipid compositions.

## Figures and Tables

**Table 1:** Comparison of the lipid compositions used (values indicate wt% and are given as indicated by the manufacturer). The symbols # and * indicate, if the lipid population originates from soy or *E. coli*, respectively.

Name	Lipid Composition
**Soybean PC**	94.6 wt% PC ^#^ + 5 wt% unknown, supplemented with 0.4 wt% DSPE-Biotin
**Soybean crude extract**	<28.6 wt% PC ^#^ + > 70.7 wt% unknown, supplemented with 0.4 wt% DSPE-Biotin
***E. coli* polar extract**	66.7 wt% PE * + 23.1 wt% PG * + 9.8 wt% CA *, supplemented with 0.4 wt% DSPE-Biotin
**PE + PC**	66.7 wt% PE ^#^ + 32.9 wt% PC ^#^, supplemented with 0.4 wt% DSPE-Biotin
**PE + PG + PC**	66.7 wt% PE ^#^ + 23.1 wt% PG ^#^ + 9.8 wt% PC ^#^, supplemented with 0.4 wt% DSPE-Biotin
**PE + PG + CA**	66.7 wt% PE ^#^ + 23.1 wt% PG ^#^ + 9.8 wt% CA *, supplemented with 0.4 wt% DSPE-Biotin

## Supplementary Materials

The following are available online at https://www.mdpi.com/1422-0067/21/19/6981/s1, Figure S1: Size distributions of the generated proteoliposomes, Figure S2: Proton turnover rate distributions for proteoliposomes made from a crude extract of soybean lipids.

## Abbreviations

CA	Cardiolipin
DPSE-PEG Biotin	1,2-distearoyl-sn-glycero-3-phosphoethanolamine-N-[biotinyl(polyethylene glycol)-2000]
DDM	n-dodecyl β-D-maltopyranoside
HPTS	8-Hydroxypyrene-1,3,6-trisulfonic acid
Ni-NTA	Nickel nitrilotriacetic acid
PMSF	Phenylmethylsulfonyl fluoride
PC	Phosphatidylcholine
PE	Phosphatidylethanolamine
PG	Phosphatidylglycerol
C*c*O	Cytochrome *c* oxidase
