# Supplementary material for: Lipid Composition Affects the Efficiency in the Functional Reconstitution of the Cytochrome c Oxidase
Source: Int J Mol Sci. 2020 Sep 23;21(19):6981. doi: 10.3390/ijms21196981 (PMC7583929; doi:10.3390/ijms21196981)
Supplement: Supplementary file 1 [file ijms-21-06981-s001.pdf]

Supplementary Material for the manuscript

Lipid Composition Affects the Efficiency in the Functional Reconstitution of the Cytochrome *c* Oxidase

Katharina Gloria Hugentobler[1], Dorothea Heinrich[2], Johan Berg[3], Joachim Heberle[4], Peter Brzezinski[3], Ramona Schlesinger[2], Stephan Block[1*]

[1] Institute of Chemistry and Biochemistry, Emmy-Noether Group "Bionanointerfaces", Freie Universität Berlin, Arnimallee 22, 14195 Berlin, Germany

[2] Department of Physics, Genetic Biophysics, Freie Universität Berlin, Arnimallee 22, 14195 Berlin, Germany

[3] Department of Biochemistry and Biophysics, The Arrhenius Laboratories for Natural Sciences, Stockholm University, SE-106 91 Stockholm, Sweden

[4] Department of Physics, Experimental Molecular Biophysics, Freie Universität Berlin, Arnimallee 22, 14195 Berlin, Germany

*Corresponding author:

Stephan Block, e-mail: stephan.block@fu-berlin.de

Table of Contents

S1. Proteoliposome size distributions 2

S2. Proton turnover rate distributions for proteoliposomes made from a crude extract of soybean lipids 3

S1. Proteoliposome size distributions

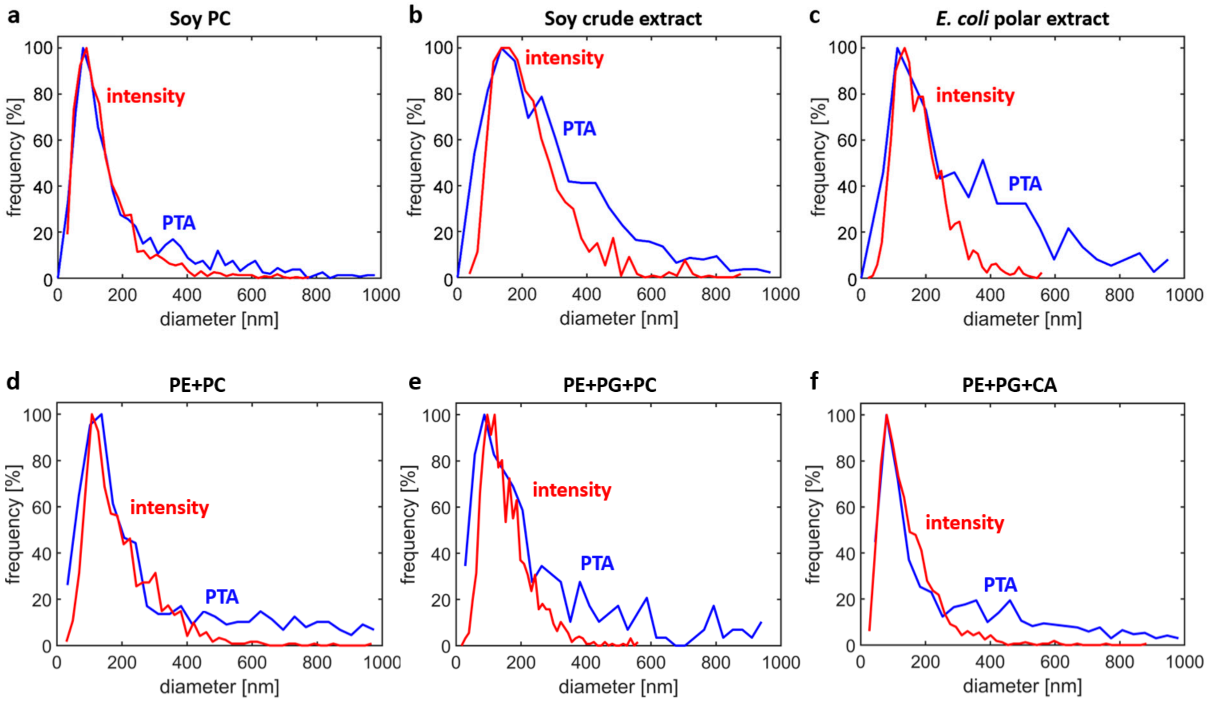

Figure S1. Size distributions of C*c*O-containing proteoliposomes which have been formed using either (a) the PC fraction of soybean-derived lipids, (b) a crude lipid fraction of soybean-derived lipid, (c) the polar extract of *E. coli* or (d-f) the "PE+PC", "PE+PG+PC", and "PE+PG+CA" mixture (as detailed in Table 1 of the main manuscript). Shown are the size distributions obtained using particle tracking analysis (PTA) of suspended proteoliposomes (blue traces) as well as using the fluorescence intensity of surface-tethered proteoliposomes (red traces; see Material and Methods section for details on these methods).

S2. Proton turnover rate distributions for proteoliposomes made from a crude extract of soybean lipids

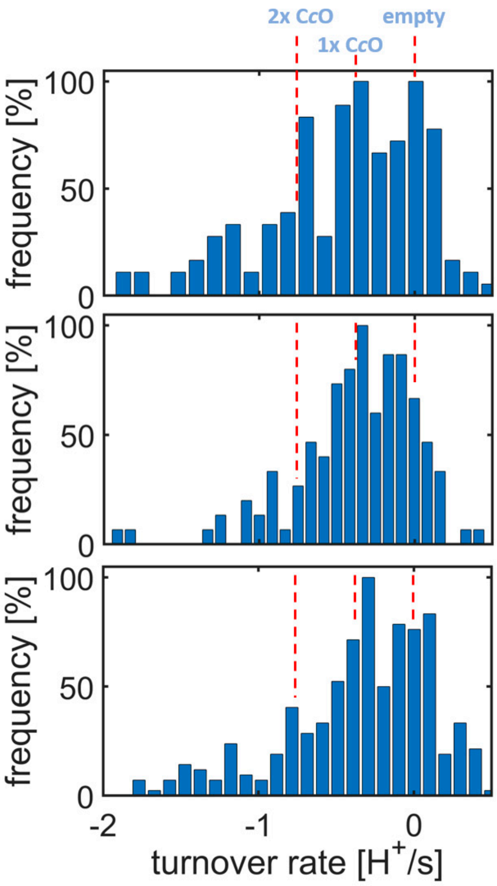

Figure S2. Distributions of the total proton turnover rate extracted from repetitions of the single-liposome assay applied to C*c*O-containing proteoliposomes (made from a crude extract of soybean lipids). These repetitions have been done on 3 independent measurement days using freshly prepared proteoliposomes. The red dashed lines indicate the position of peaks that replicate well in the repetitions.
